# *Spirocerca lupi* in the stomach of two Andean foxes (*Lycalopex culpaeus*) from Chile

**DOI:** 10.1007/s00436-023-07825-3

**Published:** 2023-04-04

**Authors:** Sophia Di Cataldo, Aitor Cevidanes, Paulina Sepúlveda-García, Mario Alvarado-Rybak, Riccardo Paolo Lia, Domenico Otranto, Karen Terio, Ananda Müller, Javier Millán

**Affiliations:** 1 Instituto de Medicina y Biología Experimental de Cuyo (IMBECU), Consejo Nacional de Investigaciones Científicas y Tecnológicas (CONICET), Mendoza, Argentina; 2 Department of Animal Health, NEIKER-Basque Institute for Agricultural Research and Development, Basque Research and Technology Alliance (BRTA), Parque Científico y Tecnológico de Bizkaia, P812, 48160 Derio, Spain; 3 Instituto de Ciencias Clínicas Veterinarias, Facultad de Ciencias Veterinarias, Universidad Austral de Chile, Valdivia, Chile; 4 Núcleo de Ciencias Aplicadas en Ciencias Veterinarias y Agronómicas, Facultad de Medicina Veterinaria y Agronomía, Universidad de las Américas, Echaurren, 140 Santiago, Chile; 5 Department of Veterinary Medicine, University of Bari, Valenzano, 70010 Bari, Italy; 6 Faculty of Veterinary Sciences, Bu-Ali Sina University, Hamedan, Iran; 7 Zoological Pathology Program, University of Illinois, Brookfield, IL USA; 8 Department of Biomedical Sciences, Ross University School of Veterinary Medicine, Basseterre, Saint Kitts and Nevis; 9 Facultad de Ciencias de la Vida, Universidad Andres Bello, República, 440 Santiago, Chile; 10 Instituto Agroalimentario de Aragón-IA2 (Universidad de Zaragoza-CITA), Miguel Servet 177, 50013 Zaragoza, Spain; 11 Fundación ARAID, Avda. de Ranillas, 50018 Zaragoza, Spain

**Keywords:** Culpeo fox, Nematoda, Spirurida, *Pseudalopex culpaeus*, South America

## Abstract

The genus *Spirocerca* includes nematodes that parasitize the stomach and the oesophagus of carnivores, chiefly canids. Herein, we provide new data about the morphological, histopathological, and molecular characterization of *Spirocerca* sp. in Andean foxes (*Lycalopex culpaeus*) in Chile. Intact immature worms, identified as *Spirocerca* sp., were recovered in the lumen of the stomach from two foxes. Histologically, worms morphologically consistent with spirurid nematodes were present within the wall of the stomach and surrounded by nodular areas of inflammation with central necrotic debris. Molecular analysis of the *cox*1 gene yielded 19 sequences and 5 nucleotide sequence types with 99.95 to 99.98% similarity, being shared between both foxes. Nucleotide similarity ranged from 93.1 (with genotype 2 of *S. lupi* and *S. vulpis*) to 95.8% (with genotype 1 of *S. lupi*), a higher similarity than noted from sequences of *S. lupi* from an Andean fox from Peru (91.0 to 93.3%). However, the Poisson Tree Processes for species delineation did not support the existence of a new species *Spirocerca*. Phylogenetic and nucleotide analyses suggest that these specimens belong to a new variant or genotype of *S. lupi* or to a cryptic species. Whether the presence of the worms in the stomach has to do with genotypic differences in parasites or host or some combination is uncertain. *Spirocerca lupi* has never been found in Chilean dogs and must be investigated.

**Supplementary Information:**

The online version contains supplementary material available at 10.1007/s00436-023-07825-3.

## Introduction

*Spirocerca* (Nematoda: Spirocercidae) is a genus of nematodes that parasitize the stomach and the oesophagus of carnivores, chiefly canids (Mazaki-Tovi et al. 2002). Within this genus, *Spirocerca lupi* is a parasite of wild and domestic canids found in tropical and subtropical regions of the world (van der Merwe et al. 2008). *Spirocerca lupi* induces the formation of nodules in the submucosa and muscular walls of the oesophagus and can migrate through the aorta, causing ossifying spondylitis of the thoracic vertebrae and aneurysms. Aberrant larval migration of this nematode into the central nervous system may cause neurological alterations (van der Merwe et al. 2008). Dogs become infected when they ingest infective third-stage larvae (L3) from an intermediate (vertebrate) or paratenic (beetle) host (Rojas et al. 2020b). *Spirocerca lupi* has also been reported parasitizing wild carnivores, as molecularly confirmed for the black-backed jackal (*Canis mesomelas*) from South Africa (Bumby et al. 2017) and the Andean fox (*Lycalopex culpaeus*) from Peru (Gómez-Puerta et al. 2018). In the latter case, *S. lupi* was found in oesophageal nodules and the aorta (Gómez-Puerta et al. 2018). Other reports on wild carnivores lack molecular confirmation (reviewed in Rojas et al. 2020a). Overall, the characterization of mitochondrial and rDNA markers revealed the existence of two well-delineated genotypes of *S. lupi*: genotype 1 from Australia, India, Israel, and South Africa and genotype 2 from Hungary and Italy (Rojas et al. 2018). The latter genotype revealed the cryptic diversity in the species and was proposed as an intermediate ‘taxon’ between *S. lupi* and *S. vulpis* (Rojas et al. 2018).

Recently another species, *Spirocerca vulpis*, was described in the red fox (*Vulpes vulpes*) (Rojas et al. 2018) and identified in several European countries (Gama et al. 2020; Martín-Pérez et al. 2020; Rojas et al. 2020b). Data suggest that *S. vulpis* is more prone to cause nodules in the stomach rather than in the oesophagus, as happens for *S. lupi* (Rojas et al. 2020b).

The Andean fox is distributed along the Andes and hilly regions of South America from the south of Colombia to Southern Chile (Jiménez and Novaro 2004). This species thrives in human-dominated landscapes, where it takes advantage of anthopogenic resources. Recently, the specimens of *Spirocerca* sp. were morphologically identified in the stomach of Andean foxes (Oyarzún-Ruiz et al. 2020). To explain this finding, different hypotheses arose about the origin of these specimens: (i) that these were *S. lupi*, as an effect of a spillover from dogs, similar to what may have occurred in the Peruvian Andean fox (Gómez-Puerta et al. 2018); (ii) that these were *S. vulpis*, or a closely related species, given the localization of both parasites in the stomach; or (iii) that these belonged to a new, yet undescribed, species for which the Andean fox is a natural host. Therefore, to provide further evidence for the hypotheses above, we provide new data about the morphological, histopathological, and molecular characterization of *Spirocerca* sp. found in Andean foxes in Chile.

## Material and methods

Two road-killed adult male Andean foxes from central Chile (Maule municipality, 35°30′55″S 71°42′06″W; Concón municipality, 32°56′42″S 71°30′48″W) were necropsied and tissues saved for histologic and parasitological evaluation. Those foxes were among the included in the survey by Oyarzún-Ruiz et al. (2020). Eleven intact worms were collected in the lumen of the stomach of one fox (i.e. fox #1) and ten specimens from fox #2. Worms were preserved in 95% ethanol until further processing. After measuring the total length of each worm, a piece of the middle part of every specimen was cut for DNA extraction. The rest of the specimens (*n*=6) from fox #2 were immersed in lactophenol solution to facilitate the observation of internal organs and their measurements. Measurements and photographs were taken using a DM-LB2 microscope and Leica Las version 4.5.0 software (Leica Microsystems, Wetzlar, Germany). Specimens from fox #1 were unfortunately lost before morphological examinations could be performed.

DNA was extracted from ten nematodes from each fox, using a DNeasy Blood & Tissue Kit (Qiagen, Hilden, Germany) according to the manufacturer’s instructions. The molecular amplification of *Spirocerca* spp. was carried out by two conventional PCR protocols (Table 1). Two percent agarose gel electrophoresis was performed, and PCR products were visualized under a UV transilluminator. All positive samples obtained were sequenced by Sanger (Macrogen Inc., South Korea), and the sequences were compared with those available in the GenBank® database.

**Table 1:** Primers used for the molecular characterization of *Spirocerca* sp. specimens from Andean foxes

Target	Primer names	Primer sequences	Fragment length (bp)	Reference
*cox*1	JB3	5′ TTTTTTGGGCATCCTGAGGTTTAT 3′	450	Bowles et al. (1995)
	JB4	5′ TAAAGAAAGAACATAATGAAAATG 3′		
*18S*	Nem 18S-F	5′ CGCGAATRGCTCATTACAACAGC 3′	750	Floyd et al. (2005)
	Nem 18S-R	5′ GGGCGGTATCTGATCGCC 3′		

For phylogenetic analyses, all sequence alignments were performed with ClustalW executed in Geneious Prime® 2021.2.2 (Biomatters Limited, 2020). Nucleotide sequence types (ntST) determination as well as nucleotide polymorphism were performed in DnaSP.6 (Rozas et al. 2017). Phylogenetic relationships among our sequences and others submitted in the GenBank® database were assessed with a maximum likelihood tree with 1000 bootstrap replicates using the software MEGA 7.0.26 (Kumar et al. 2016), for which the best model, Tamura-Nei, was selected with jModelTest 2.1.6 (Darriba et al. 2012). To infer genetic relationships, a median-joining network was constructed using PopART (Bandelt et al. 1999), using the same sequences from the tree, and with a connection limit of 95%.

The Poisson Tree Processes (PTP) (Zhang et al. 2013) for species delimitation were used to identify the most likely number of species present in the samples using the *cox*1 gene sequences. The input Newick file was generated in Mega 7.0.26 from the ML phylogenetic tree used in the analysis described above. The algorithm was implemented on the website with 100,000 MCMC generations, a ‘burn-in’ length of 0.1, and a thinning of 100.

Representative sections of organs collected at necropsy were fixed in 10% neutral-buffered formalin and processed routinely for histopathology. Sections (2.5μm) were stained with haematoxylin-eosin and reviewed by a board-certified veterinary pathologist (KAT).

The new sequences obtained were submitted to GenBank® with the accession numbers OP476447-OP476451 (*cox*1) and OQ359120 (*18S*).

## Results

### Location and anatomopathological lesions

**Fig. 1:**
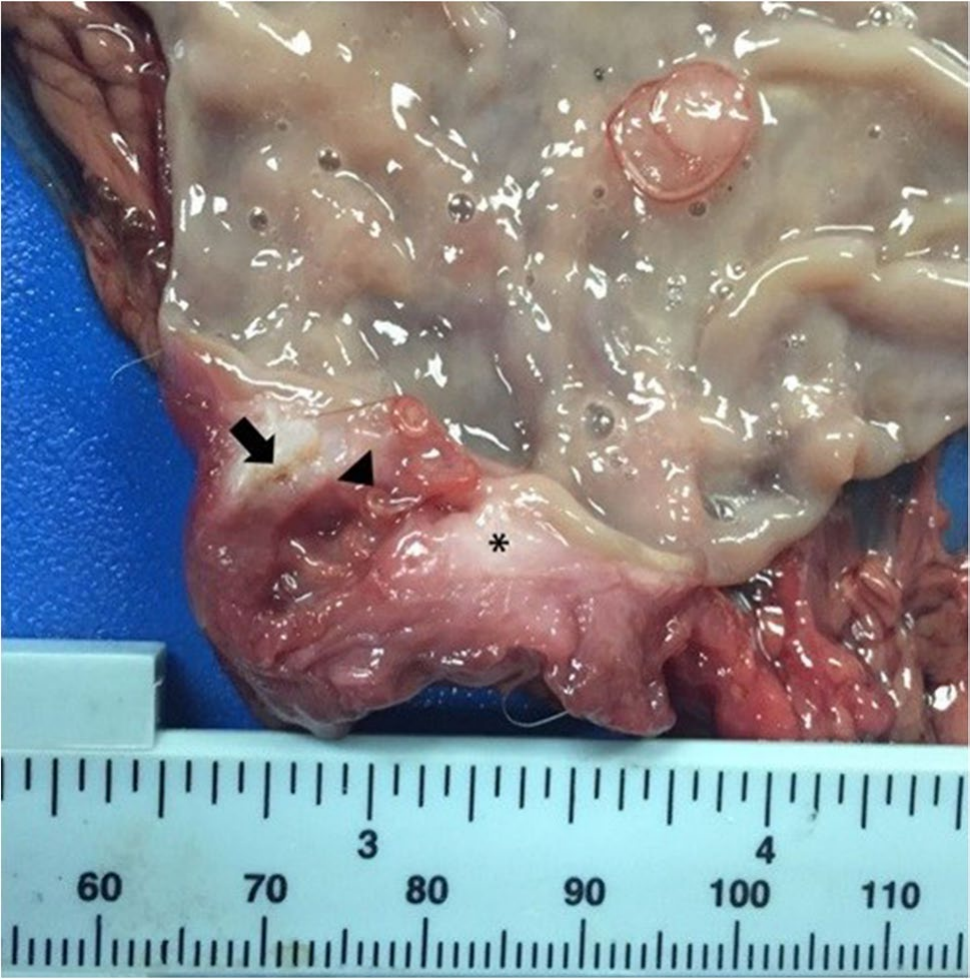
Cross section of a nodule in the fundic region of the stomach. A granulomatous tissue base (asterisk) is observed, which surrounds *Spirocerca lupi* (arrow head). In addition, in the fibrous tissue, it is possible to observe the presence of small nematode cysts (arrow)

A nodular structure (1 × 0.5 cm) was observed in the stomach fundus of fox #1, with a granulomatous and necrotic tissue forming an ulcer in the mucosa where eleven worms were recovered (Fig. 1). In fox #2, two dark-coloured nodules were observed, one of 0.5 × 0.5 cm and the other of 0.7 × 0.5 cm, both close to the pylorus and the greater stomach curvature. Ten specimens were retrieved from this fox.

**Fig. 2:**
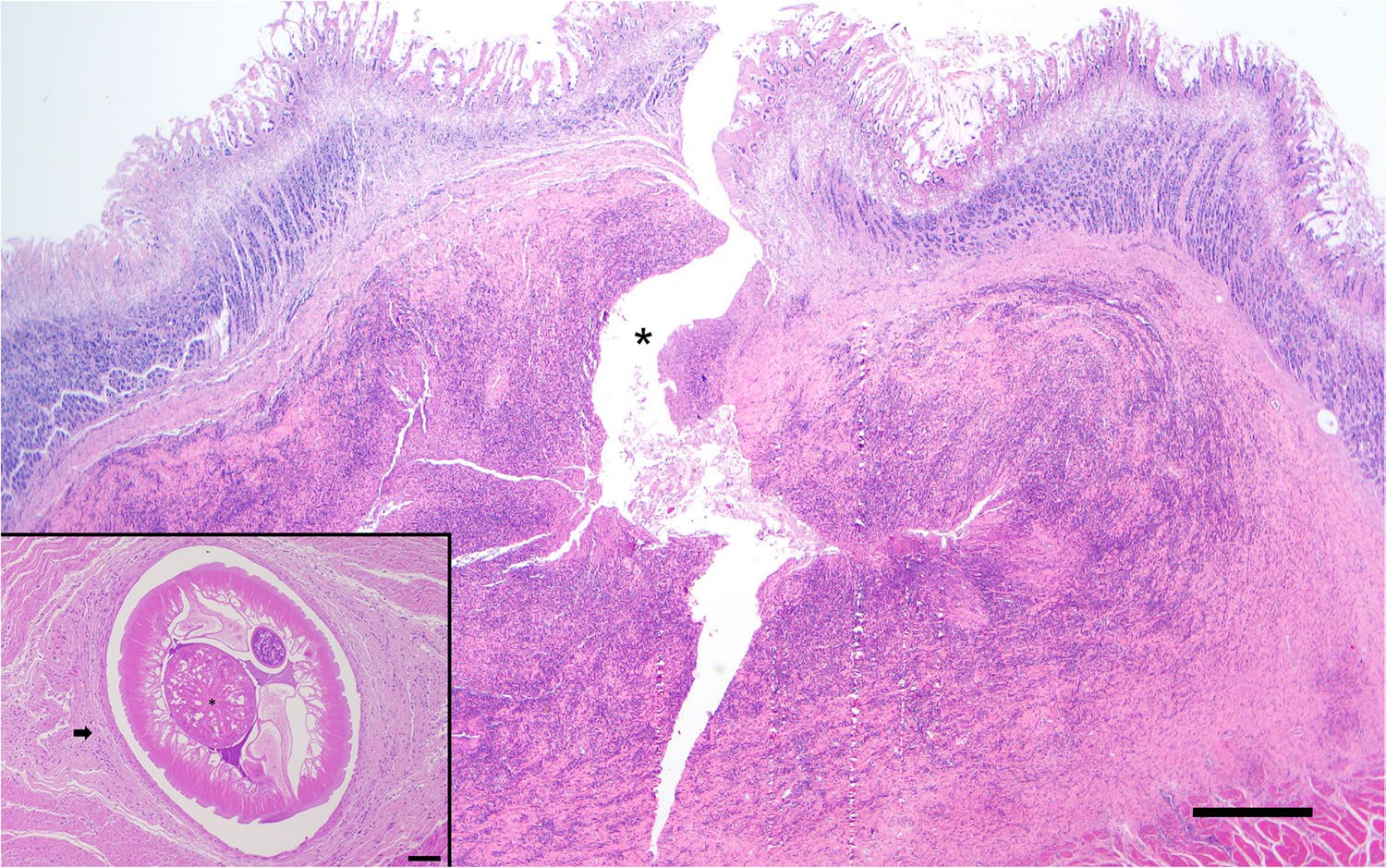
Histologic section of the stomach with inflammatory nodule that raises the overlying mucosa and displaces the muscularis. Within the centre of the inflammatory nodule, there is central cavitation (asterisk) with necrosis consistent with a necrotic parasitic track. Haematoxylin-eosin stain. Bar = 1 mm. Inset is a histologic section of a nematode surrounded by fibrous connective tissue (arrow). The nematode (a female) has cuticular bosses, coelomyarian musculature, lateral chords, prominent brush border (asterisk), and reproductive tract. Haematoxylin-eosin stain. Bar = 90 mm

Histological lesions in both fox were similar. Within the submucosa, there was a large (up to 6 mm diameter) poorly demarcated nodular accumulation of inflammatory cells surrounding central cavitation and necrotic debris (Fig. 2). These nodules raised the overlying mucosa and displaced the muscularis and disrupted submucosal collagen. Inflammatory cells were predominately lymphocytes and plasma cells with numerous plump immature fibroblasts. Neutrophils and macrophages surrounded central necrotic debris. Inflammatory cells extended into the adjacent muscularis. In areas with more chronic inflammation, there is evidence of immature granulation tissue. Within necrotic debris were brightly eosinophilic fragments of foreign material consistent with degenerate fragmented parasites as well as intact parasites. Transversal sections of the parasites were approximately 600 microns in diameter, with cuticular bosses, coelomyarian polymyarian musculature, lateral chords, the intestine with a prominent brush border, and the reproductive tract (ovary and uteri). Small amounts of fluid were also present within the pseudocoelom.

### Morphology of collected specimens

Specimens were reddish when collected. All anatomical measurements (Tables 2 and 3; Supplementary File 1) from the six specimens were compatible with *S. lupi* (Segovia et al., 2001; Vicente et al., 1997; Skrjabin et al., 1967).

**Table 2:** Measures of five *Spirocerca* sp. females from an Andean fox, Chile

	n°1 ♀	n°2 ♀	n°3 ♀	n°4 ♀	n°5 ♀	Mean ♀	Mean *S. vulpis* (Rojas et al. 2018)	Mean *S. lupi* (Rojas et al. 2018)	Mean *S. lupi* from Peruvian Andean fox (Gómez-Puerta et al. 2018)
Body length (cm)	1.360	-	1.305	2.320	2.150	1.782	6.335	6.234	6.5
Body width (μm)	404.4	439.1	493.4	477.6	559.8	474.8	1110	1034	908
Tail length (μm)	126.3	-	-	137.5	-	131.9			
Stoma diameter (μm)	85.8	73.2	75.5	105.6	72.9	82.6	84.0	100.0	102.0
Buccal capsule length (μm)	65.8	55.9	67.1	69.5	63.7	64.4	98.0	114.0	102.0
Buccal capsule width (μm)	25.7	26.3	21.6	36.7	53.8	32.82	171.0	185.0	65.0
Nerve-ring from anterior extremity (μm)	167.2	197.5	138.8	209.5	247.7	192.14	560.0	543.0	
Excretory pore from anterior extremity (μm)	226.7	310.9	222.0	-	277.5	259.275	608.0	715.00	
Oesophagus length (μm)	3240	3290	3300	3430	3590	3370	6580	6920	6980
Muscular portion length (μm)	358.4	281.9	360.6	283.6	253.3	307.56	416.0	670.0	531.0
Muscular portion width (μm)	85.9	62.0	64.4	80.1	95.0	77.48			137.0
Glandular portion length (μm)	2880	3010	3080	3050	3330	3070	6170	6250	6449
Glandular portion wide (μm)	221.4	192.7	225.5	288.3	217.8	229.14			439.0
% Oesophagus to the total body length				14.81	16.70	15.76	9.934	11.328	9.921
Vulva from anterior extremity (μm)	1347	1739	1352	1527	1795	1246	9772	2036	4224

**Table 3:** Measures of a *Spirocerca* sp. male from an Andean fox, Chile

	n°1 ♂	Mean *S. vulpis* (Rojas et al. 2018)	Mean *S. lupi* (Rojas et al. 2018)	Mean *S. lupi* from Peruvian Andean fox (Gómez-Puerta et al. 2018)
Body length (cm)	2.01	3.96	3.65	4.0
Body width (μm)	225.3	690	739	726
Caudal alae length (μm)	924.6			
Caudal alae wide (μm)	118.2			
Left spicule length (μm)	3019	2364	2216	2698
Left spicule wide (μm)	20.2			
Right spicule length (μm)	651.3	566	605	697
Right spicule wide (μm)	40.8			
Gubernaculum length (μm)	59.2			
Tail length (μm)	787.3			

#### Females

A total of five female nematodes were analysed morphologically. The female nematodes were 1.30–2.32 cm (mean 1.78 cm) in total body length with a width of 474.8 μm (404.4–493.4 μm) at the oesophagus-intestinal junction. The anterior part has a hexagonal opening and six pseudo-lips with six cervical papillae, four cephalic papillae, and two amphids. The stoma diameter was 72.9–105.6 μm (82.6 μm), and the sclerotized buccal capsule measured 55.9–69.5 μm (64.4 μm) in length and 21.6–53.8 μm (32.8 μm) in width. The oesophagus was divided into an anterior muscular part, short, and posterior glandular portion, long. The total length of the oesophagus was 3.24–3.59 mm (3.37 mm).

#### Male

Only the posterior end of one male was analysed. The posterior end was ventrally curved, with copulatory organs arranged in a caudal alae, a parallel longitudinal cuticular striations, and two spicules unequal in length and shape. In ventral view, six pairs of pedunculated papillae are present, of which four are preanal and two pairs are postanal. The distance between preanal papillae 1 and 2, 2 and 3, and 3 and 4 were 46.64 μm, 63.49 μm, and 33.14 μm, respectively. The two papillae postanal are nipple-shaped and of different sizes, the first one with a shorter peduncle. The distance between postanal papillae 1 and 2 is 156.74 μm. Gubernaculum present is shaped irregularly (triangular) and visible at the extremity of the tail. There is a single large median preanal papilla 45.378 μm wide between the last pre-cloacal papilla and the first post-cloacal papilla. The paired spicules were unequal in size with the left one being longer, thinner (needle shape), and mildly sclerotized. The greater spicule is 3.019 mm long and presented with the knobbed distal end, 41.191 μm wide and 89.843 μm long shaped like a tongue. The distal portion forms a wide curve, folding back on itself and continuing along its entire length. This description would seem atypical in this specimen, as the descriptions of other authors report a greater spicule without the formation of the curve. The greater spicule ends in a point at the level of the middle of the shorter spicule flanking it, measuring 22.6–24.6 μm (23.4 μm) wide. The right spicule is 651.39 μm long with a distal portion, which is broader and rounder than the base, with a width of 40 μm and ends with a rounded tip. The distance of the cloaca from the distal end is 365.568 μm.

### Molecular analyses

#### 18S gene

**Fig. 3:**
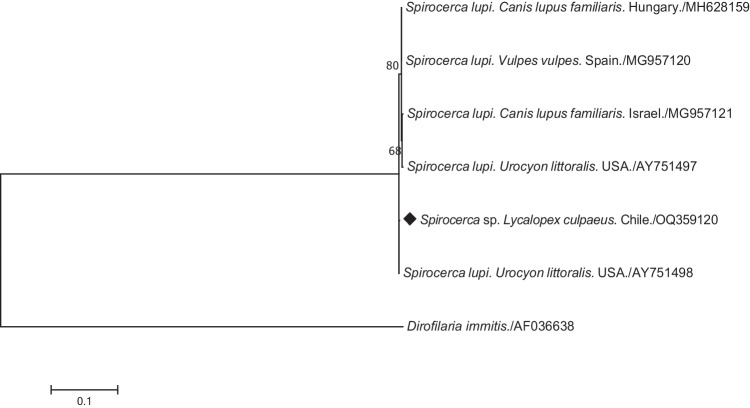
Maximum likelihood tree of the 18S rRNA gene (750 bp) of *Spirocerca* species from canids. The diamond marks the nucleotide sequence type (ntST) from our study

Twenty readable sequences were obtained, and only one nucleotide sequence type (ntST) was identified among our sequences. This ntST showed 99.9% identity with a *Spirocerca* sp. from an Island fox (*Urocyon littoralis*) in the USA (AY751498). Phylogenetic analyses of the 18S gene grouped our ntST in a branch with *S. lupi* sequences from other canids of the world (Fig. 3).

#### cox1 gene

Nineteen readable sequences were obtained, and five ntST were identified. ntST-1 was shared by 15 specimens, including eight from fox #1 and seven from fox #2. The other four ntST (2 to 4) corresponded to one specimen each, two from each fox. These five sequences showed between 99.95 and 99.98% similarity among them, 95.8% (mean=94.5% ± standard deviation (SD)=1.13) with the closest sequence of genotype 1 of *S. lupi* (MH634010, dog, India), 93.1% (93.05% ± 0.17) with genotype 2 of *S. lupi* (MH634012, dog, Hungary), and also 93.1% (92.4% ± 0.54) with the closest sequence of *S. vulpis* (MH633991, red fox, Spain). The similarity with the sequences of *S. lupi* from the Andean fox from Peru ranged from 91.0 to 93.3%. *Spirocerca* spp. sequence polymorphism of our sequences from the *cox*1 gene revealed a *Hd* of 0.386 (SD= 0.139), a *Pi* of 0.00127 (SD= 0.00051), and a *k* of 0.421 between specimens.

**Fig. 4:**
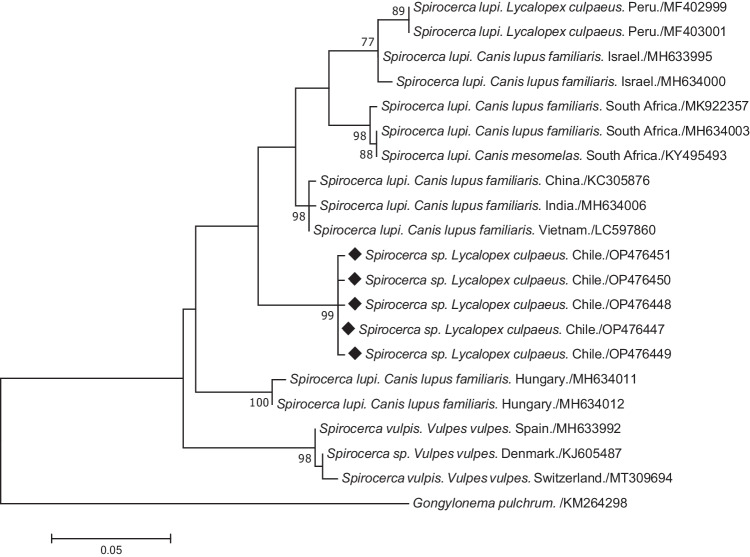
Maximum likelihood tree of the *cox*1 gene (450 bp) of *Spirocerca* species from canids all around the world. Diamonds mark the five nucleotide sequence types (ntST) detected in foxes from this study

**Fig. 5:**
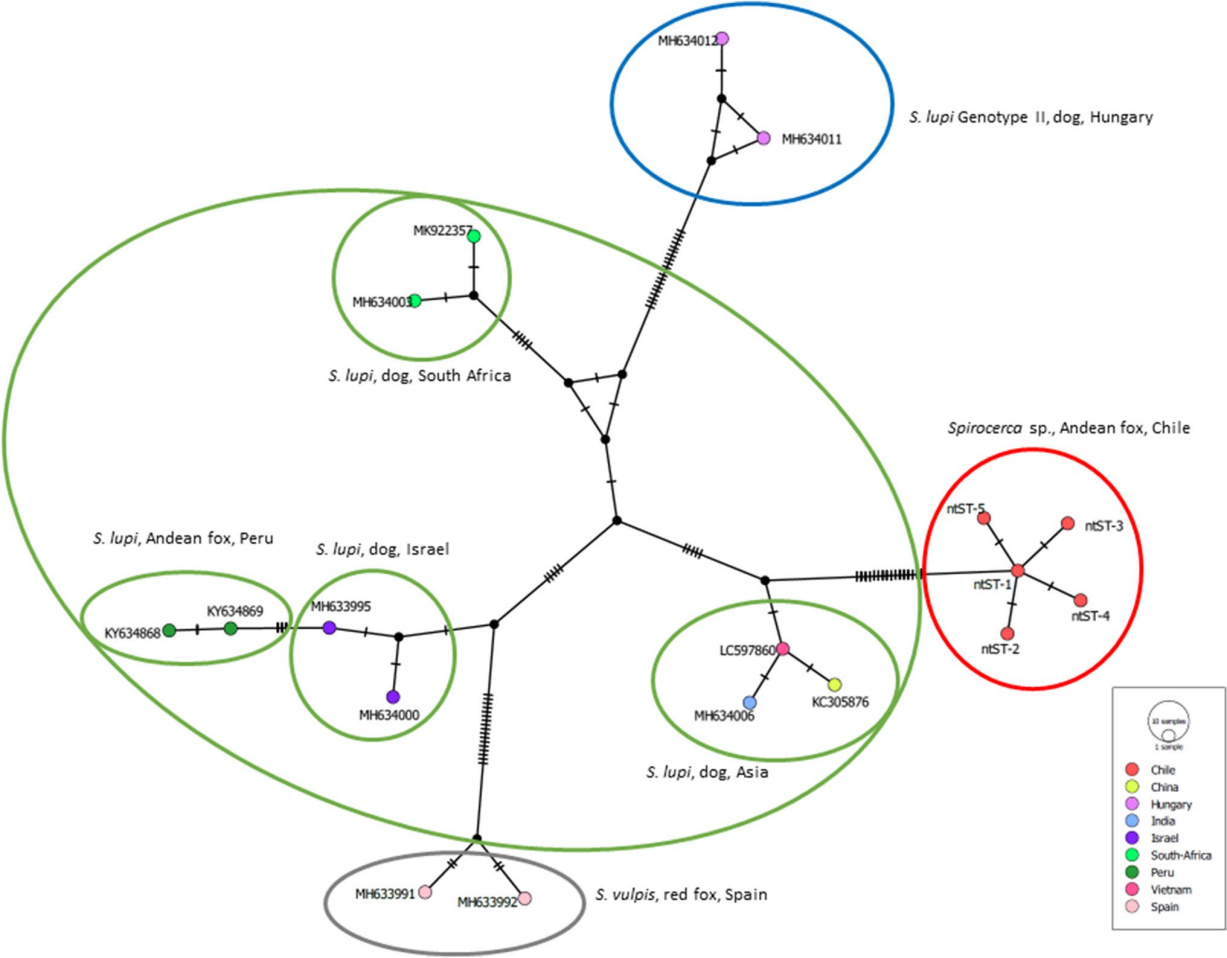
Median-joining network of the *cox*1 gene (450 bp) of *Spirocerca* species from canids all around the world. Each circle in the networks corresponds to a different nucleotide sequence type (ntST), and the size of the circles corresponds to ntST frequencies. The colour of the circles corresponds to the country where the ntST was detected. The coloured ellipses group *Spirocerca* species by geographical region and/or host species. The hatch marks indicate the number of mutations. The black circles correspond to the nodes that separate the sequences

Phylogenetic analyses grouped all the obtained sequences in a branch with *S. lupi* genotype 1 sequences (Fig. 4). Sequences belonging to genotype 2 of *S. lupi* were classified in a separate branch. Network analysis confirmed this classification, showing numerous substitution nucleotides between ours and other *Spirocerca* spp. sequences (Fig. 5). Sequences from *S. lupi* genotype 2, *S. vulpis,* and our sequences were positioned in the extremes of the network.

The PTP analysis determined three putative species in the complete *Spirocerca* spp. database according to the maximum likelihood and Bayesian solutions: (i) *S. lupi* genotype 1 (support > 0.936); (ii) *S. lupi* genotype 2 (support > 0.829); and (iii) *S. vulpis* (support > 0.949). This analysis did not support a division within genotype 1 of *S. lupi*. There was no support for the specimens from the Chilean Andean foxes, which were grouped in the *S. lupi* genotype 1 group, belonged to a separate species (support = 0.426).

## Discussion

Data presented support the existence of a putative new variant of *S. lupi* parasitizing the Andean fox in Chile. The nodules induced by the parasites were in the stomach in both foxes. *Spirocerca lupi* is more commonly located in the oesophagus, the aorta, or the lung, even in wild canids (see the review in Rojas et al. 2020a). This pointed towards the possibility of the parasites being *S. vulpis*, which is typically located in the stomach of red foxes (Rojas et al. 2020a), as observed in the present cases. However, molecular, phylogenetic, and network analysis clearly indicates that our specimens do not share identity with *S. vulpis*. In addition, it seems unlikely that two distinct fox species with such distant distribution areas would share the same parasite species.

The molecular characterization of the *cox*1 fragment indicates a genetic distance, which is in the line with the existing between *S. lupi* and *S. vulpis*. These two species presented pairwise nucleotide differences of about 6.5% in the *cox*1 locus (Rojas et al. 2018), whereas our sequences showed differences of up to about 4.2% with *S. lupi* (genotype 1), 6.4% with *S. lupi* (genotype 2), and 6.9% with *S. vulpis*. The differences between both genotypes of *S. lupi* were 5.5% (Rojas et al. 2018). However, PTP analyses did not support the existence of a new species, although this analyses must be taken with caution because the tree used as input had support under 70% in the internal nodes. As such, Andean foxes would host at least, and according to the phylogenetic and network analyses, a new variant or genotype of *S. lupi* or a cryptic species (see Cháves-González et al. 2022). Based on the above, it is surprising that the location of the specimens was in the stomach and not in the oesophagus. It is also surprising why this parasite was never diagnosed in dogs in Chile, given that *S. lupi* has been found in dogs in other South American countries (see review by Rojas et al. 2018). This can be due to a lack of surveillance, but, given that this nematode is conspicuous, especially pathogenic (Mazaki-Tovi et al. 2002), and can be easily diagnosed in live dogs (van der Merwe et al. 2008) this is unlikely. Perhaps the parasite is in an early stage of introduction in Chile and is present only in rural regions, where dog owners rarely take their dogs for veterinary assistance. The geographical isolation of Chile often explains differences in distribution in parasites with neighbouring countries (Di Cataldo et al. 2022), but then the question regarding how *S. lupi* got to parasitize Andean foxes remains unanswered. The closest sequences, more similar than the sequences from the Peruvian Andean fox, were those from Asiatic specimens of *S. lupi*, so perhaps the parasite arrived in Chile through a Pacific Ocean route (airway or via maritime transport) more than from a terrestrial invasion from a neighbour country. The latter, as mentioned, is difficult given the geographic barriers (high plateaus, deserts, Andean mountains) that separate Chile from the rest of the region. Given the high nucleotide divergence of the specimens collected from Andean fox from Chile and Peru (belonging to the genotype 1), they may have evolved independently.

The histologic lesions noted in the stomach are similar to those previously reported for other canids parasitized with *Spirocerca* and consistent with early inflammatory nodules although they lacked adult worms (Dvir et al. 2020). Notably, no lesions were identified in more common sites such as the aorta or oesophagus, although only oesophagus was evaluated histologically. While the stomach has been previously reported site of infection, the absence of concurrent lesions in these other sites is unusual. In the previous report of infection in a Peruvian Andean fox, aortic lesions and aneurysms were associated with the nematode (Gómez-Puerta et al. 2018). In maned wolves (*Chrysocyon brachyurus*) from Brazil, *S. lupi* was also associated with a granulomatous pneumonia (Blume et al. 2014) and, again, no lesions were noted in the examined sections of the lung from these Andean foxes. Whether the differences in location have to do with genotypic differences in parasites or host or some combination is uncertain. There was no evidence of neoplastic transformation in either case.

Unfortunately, the only specimens we were able to examine morphologically and histopathologically were immature individuals, which prevented comparison of organ size with specimens from other species. However, the distal portion of the greater spicule of the male showed an atypical feature. Nevertheless, considering the type of host, the location of the parasites, and the phylogenetic and network analysis based on a fragment of the *cox*1 gene, our results are highly suggestive of the existence of, at least, a new genotype of *S. lupi*. Besides, our study confirms the high variability of the parasites in its *cox*1 gene, even considering that most specimens were siblings, with five different haplotypes among 19 individuals from the two hosts, in agreement with previous observations in *S. lupi* and *S. vulpis* (Rojas et al. 2018). We also confirmed that, in contrast, the analysis of the 18S gene does not provide an accurate phylogenetic differentiation of the diverse *Spirocerca* species, as previously observed (Rojas et al. 2018).

In summary, these findings prompt additional questions including why nodules were found in the stomach and what the prevalence and epidemiology of *Spirocerca* is in Chile and elsewhere in South America. The morphological analysis of adult specimens, further molecular characterization, collection and analysis of potential intermediate hosts, and systematic helminthological studies in dogs will be necessary to answer these questions.

## Supplementary information

ESM 1

## Data Availability

Not applicable.
