# Supplementary material for: Spirocerca lupi in the stomach of two Andean foxes (Lycalopex culpaeus) from Chile
Source: Parasitol Res. 2023 Apr 4;122(6):1261–9. doi: 10.1007/s00436-023-07825-3 (PMC10172281; doi:10.1007/s00436-023-07825-3)

**Supplementary File.**

**Macroscopic photographs of *Spirocerca* sp. from Andean foxes, Chile**

**Fig S1:** Male (partial nematode)

**Male:** Examination of the posterior end **(Fig. 1a)**. The posterior end is ventrally curved, with copulatory organs **(Fig. 1b)** provided of caudal alae (arrow) with parallel longitudinal striations (arrowhead). In ventral view are shown six pairs of pedunculated papillae **(Fig. 1c)**, of which four are preanal (arrow) and two are postanal (arrowhead). These latter are nipple-shaped and the first is characterized by a shorter peduncle. The distance between preanal papillae 1 and 2, 2 and 3, and 3 and 4 are 46.640 µm, 63.498 µm and 33.145 µm, respectively. The distance between postanal papillae 1 and 2 is 23.4 µm. The *Gubernaculum* (arrow) of irregular (triangoliformes) shaped is visible at the extremity of the tail **(Fig. 1d).**


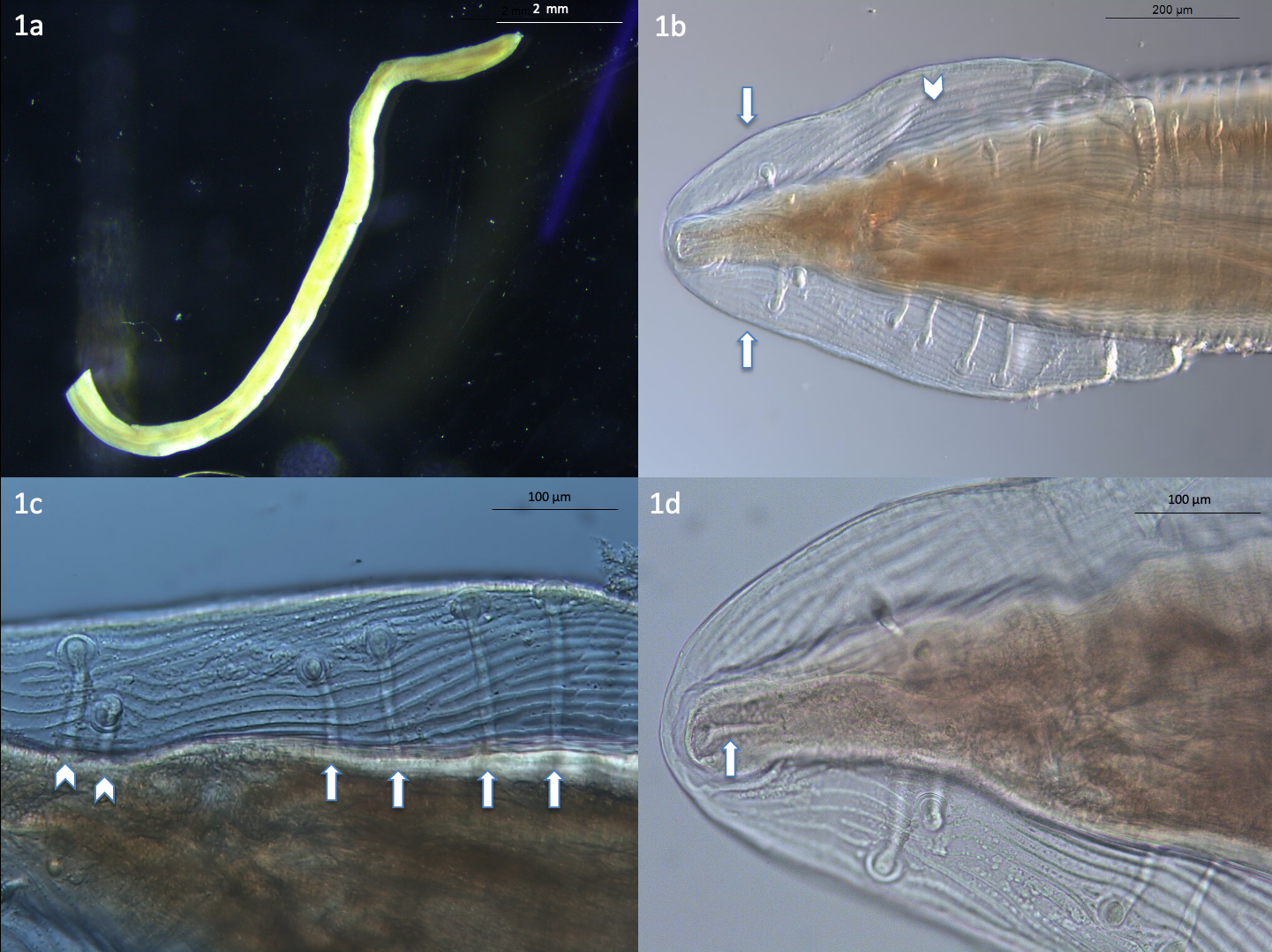


**Fig S2:** Posterior end of the male.


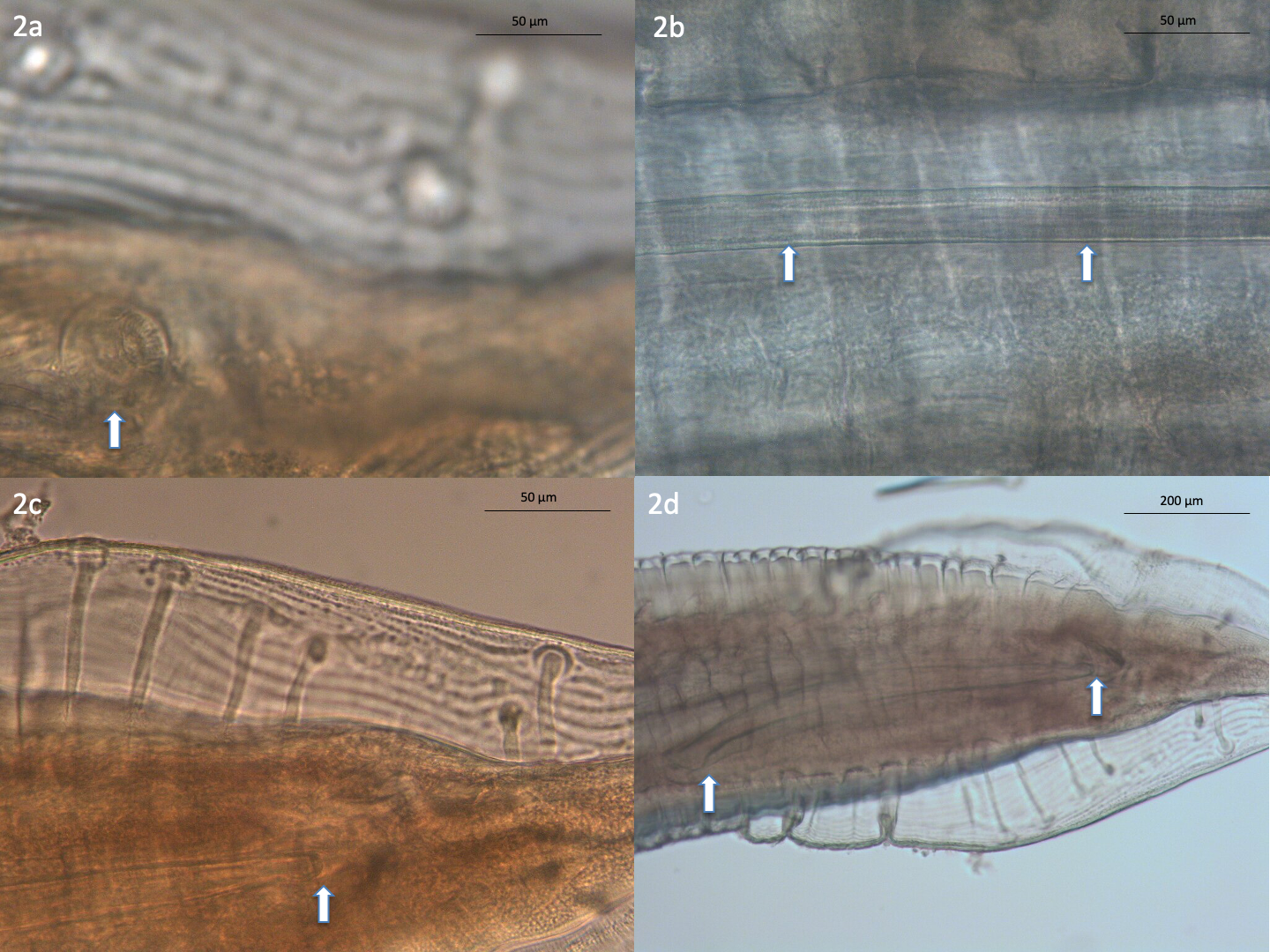
Ventrally, the posterior end is characterized by a single large **(Fig. 2a)** median preanal papilla (arrow) of 45.378 µm wide, localized between the last pre cloacal papilla and the first post cloacal papilla. The paired spicules are unequal with the left one being longer, thinner (needle shape) and mildly sclerotized **(Fig. 2b)**. The greater spicule is 3.019 mm long and presents a knobbed proximal end, which is 41.191 µm wide and 89.843 µm long. The distal end is tapered **(Fig. 2c)**. The right spicule **(Fig. 2d)** is 651.393 µm long with a broader and rounder proximal end and a rounded tip. The distance between the cloaca and the distal end is 365.568 µm.

**Fig S3:** Head of the female.

**Females:** The anterior part has a hexagonal opening and six pseudo-lips **(Fig. 3a)** with cervical papillae (arrow). The stoma **(Fig. 3b)** diameter was 73.5-69.5 µm (64.5 µm), the sclerotized buccal capsule (arrow) measured 55.9-105.6 µm (64.5 µm) long and 21.6-36.7 µm (27.5 µm) wide. The oesophagus-intestinal junctions were 453.6 µm (404.4-493.4 µm) distant from the anterior end. The total length of the oesophagus was 3.360.5 µm (3.344-3.591 µm). The muscular oesophagus **(Fig. 3c)** measured 283.6 µm (253.3-360.6 µ) in length and 77.84 µm (62.0-95.0 µm) in width. The glandular oesophagus **(Fig. 3d)** measured 3.075 µm (2.885-3.388 µm) long and 231.9 µm (192.7-288.3 µm) wide, with a ratio of the glandular-muscular oesophagus of 1:11.


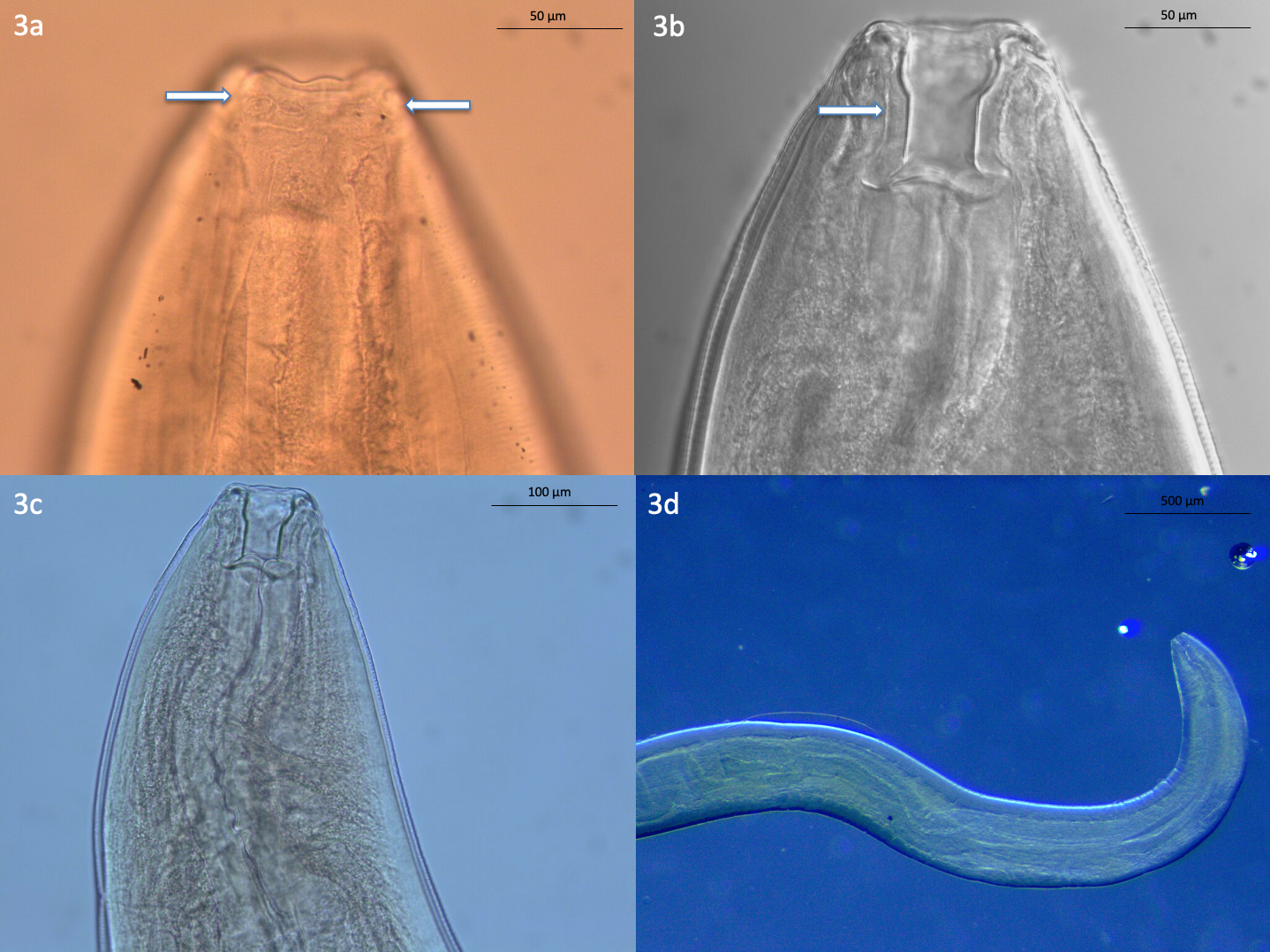


**Fig S4:** Excretory pore, vulva and posterior of female.

The distance of the excretory pore **(Fig. 4a)** from the proximal end was 192.1 µm (138.8-209.5 µm). Vulva was at the level of oesophagus 760 µm from the anterior end **(Fig. 4b)**. The distance of the anus opening from distal end was 123.3 µm **(Fig. 4c)** and the posterior end has a conic tail accompanied by terminal papilla **(Fig. 4d)**.


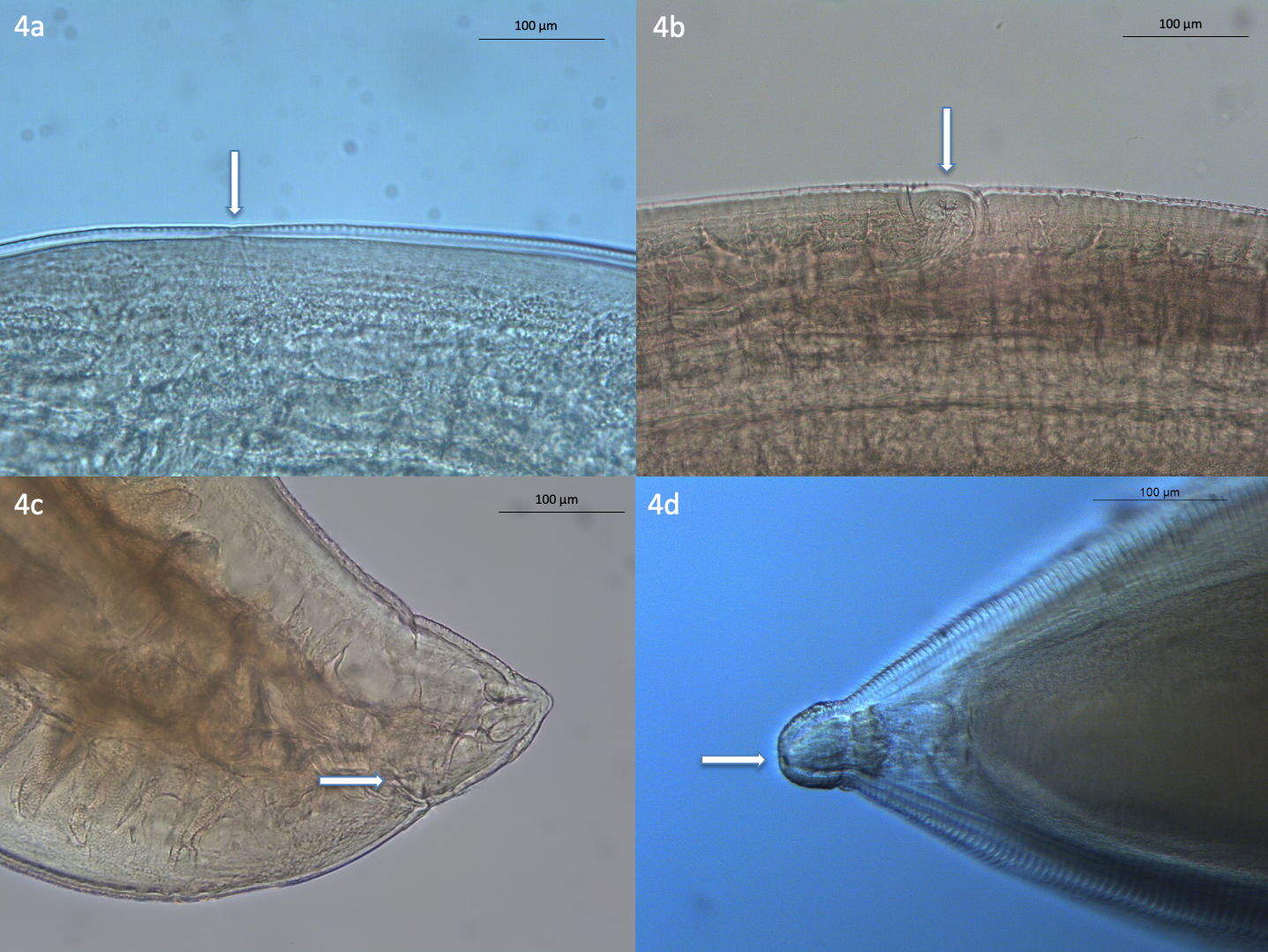

Supplement: Supplementary file 1 — ESM 1 [file 436_2023_7825_MOESM1_ESM.docx]
